# An Invitation to Greater Use of Matthews Correlation Coefficient in Robotics and Artificial Intelligence

**DOI:** 10.3389/frobt.2022.876814

**Published:** 2022-03-25

**Authors:** Davide Chicco, Giuseppe Jurman

**Affiliations:** ^1^ Institute of Health Policy Management and Evaluation, University of Toronto, Toronto, Canada; ^2^ Data Science for Health Unit, Fondazione Bruno Kessler, Trento, Italy

**Keywords:** Matthews correlation coefficient, accuracy, F_1_ score, binary classification, machine learning, robotics, artificial intelligence

## 1 Introduction

A binary classification is a computational procedure that labels data elements as members of one or another category. In machine learning and computational statistics, input data elements which are part of two classes are usually encoded as 0’s or –1’s (negatives) and 1’s (positives). During a binary classification, a method assigns each data element to one of the two categories, usually after a machine learning phase. A typical evaluation procedure then creates a 2 × 2 contingency table called *confusion matrix*, where the positive elements correctly predicted positive are called *true positives* (TP), the negative elements correctly predicted negative are called *true negatives* (TN), the positive elements wrongly labeled as negatives are called *false negatives* (FN), and the negative elements wrongly labeled as positives are called *false positives* (FP).

Since it would be difficult to always analyze the four categories of the confusion matrix for each test, scientists defined statistical rates that summarize TP, FP, FN, and TN in one value. Accuracy (Eq. 1), for example, is a rate that indicates the ratio of correct positives and negatives (Zliobaite, 2015), while F_1_ score (Eq. 2), is the harmonic mean of positive predictive value and true positive rate (Lipton et al., 2014; Huang et al., 2015).

$$\text{accuracy}=\frac{TP+TN}{TN+TP+FP+FN}$$ (1)

(worst value = 0; best value = 1).

$${\text{F}}_{1}\;\text{score}=\frac{2\cdot TP}{2\cdot TP+FP+FN}$$ (2)

(worst value = 0; best value = 1).

Even if accuracy and F_1_ score are very common in machine learning studies, they can be misleading (Chicco and Jurman, 2020) in several situations.

The Matthews correlation coefficient (Eq. 3) (Matthews, 1975), instead, is the only statistical rate that generates a high score only if the values of the four basic rates (sensitivity, specificity, precision, negative predictive value) are high (Yao and Shepperd, 2020; Zhu, 2020). For this reason, the MCC results being more informative and reliable than accuracy, F_1_ score, and many other rates (Jurman et al., 2012; Chicco, 2017; Chicco and Jurman, 2020; Chicco et al., 2021; Chicco et al., 2021a; Chicco et al., 2021b).

$$\text{MCC}\;=\frac{TP\cdot TN-FP\cdot FN}{\sqrt{\left(TP+FP\right)\cdot \left(TP+FN\right)\cdot \left(TN+FP\right)\cdot \left(TN+FN\right)}}$$ (3)

(minimum value = −1; maximum value = +1).

where MCC = +1 means perfectly correct prediction (all the positives correctly predicted positives and all the negatives correctly predicted negatives), MCC = 0 means the prediction was no better than random guessing, and MCC = –1 means perfectly wrong prediction (that is, all the ones were predicted zeros and all the zeros were predicted ones).

Despite the large usage of the MCC in machine learning, bioinformatics, and health informatics, we decided to investigate how popular this rate was in robotics and artificial intelligence.

## 2 Analysis

**Method**. To investigate the usage of these three confusion matrix rates in robotics and artificial intelligence, we performed a search of the Matthews correlation coefficient, accuracy, F_1_ score keywords in ten preeminent scientific journals on robotics. We counted the number of publications containing each keyword, per each journal, through Google Scholar. For example, we used the following search terms on Google Scholar to count the number of articles containing the “Matthews correlation coefficient” keyword in the *Frontiers in Artificial Intelligence* journal:

“Matthews correlation coefficient” source:“Frontiers in Artificial Intelligence”

We performed this search for ten robotics journals (*Frontiers in Artificial Intelligence, Robotics and Autonomous Systems, Frontiers in Neurorobotics, International Journal of Robotics Research, Journal of Field Robotics, Frontiers in Robotics and AI, IEEE Robotics and Automation Letters, IEEE Transactions on Robotics, Science Robotics, Journal of Intelligent and Robotic Systems*) and reported the results in Table 1.

**TABLE 1:** Occurrences of the keywords in the articles of the journals. #MCC: number of articles containing the “Matthews correlation coefficient” keyword for each journal. #accuracy: number of articles containing the “accuracy” keyword for each journal. #F_1_ score: number of articles containing the “F1 score” keyword for each journal. We did all the searches on 14 February 2022 at 2:00p.m. EST, by using the source keyword on the Google Scholar search field at https://scholar.google.com We sorted the scientific journals alphabetically.

Scientific Journal	#MCC	#Accuracy	#F_1_ Score
Frontiers in Artificial Intelligence	6	324	28
Frontiers in Neurorobotics	1	439	16
Frontiers in Robotics and AI	0	596	14
IEEE Robotics and Automation Letters	0	2,390	71
IEEE Transactions on Robotics	0	1,750	8
International Journal of Robotics Research	1	1,540	10
Journal of Field Robotics	1	688	12
Journal of Intelligent and Robotic Systems	0	1,010	13
Robotics and Autonomous Systems	5	1,900	21
Science Robotics	0	135	0
Average	1.4	1,077.2	19.3
Median	0.5	849	13.5
Range	[0; 6]	[135; 2,390]	[0; 71]

**Results**. As we can see in the table indicating the number of articles including each keyword per each robotics journal (Table 1), the MCC was employed in very few articles among all the journals. *Frontiers in Artificial Intelligence* had the highest number, six, while *Robotics and Autonomous Systems* had five. Only one article published in *Frontiers in Neurorobitics*, *International Journal of Robotics Research*, and *Journal of Field Robotics* each contained results measured by the Matthews correlation coefficient. No article mentioning the MCC was found in the other five journals (*Frontiers in Robotics and AI*, *IEEE Robotics and Automation Letters*, *IEEE Transactions on Robotics*, *Robotics and Intelligent Systems*, and *Science Robotics*). The average number of articles including MCC results in these ten journals is 1.40 (Table 1).

On the contrary, we found hundreds and thousands of articles mentioning the accuracy rate (Table 1), ranging from 135 articles of *Science Robotics* to 2,390 studies published in *IEEE Robotics and Automation Letters*. The average number of articles including accuracy results in these ten journals is 1,077.2 (Table 1).

The number of articles including the F_1_ score was smaller than the accuracy ones, but definetely more than the MCC studies. The number of F_1_ score articles ranged from none (*Science Robotics*) to 71 (*IEEE Robotics and Automation Letters*), with an overall average value of 19.30 (Table 1). Almost all the journals had at least ten published articles containing results measured by F_1_ score, except *IEEE Transactions on Robotics* with eight articles and the already mentioned *Science Robotics* with zero.

## 3 Discussion

Our results clearly show that the Matthews correlation coefficient is almost unknown in robotics. F_1_ score is clearly underused with respect to accuracy, but it is still known for all the journals except *Science Robotics*. The MCC, instead, is clearly out of radar for most of the robotics researchers that published articles in these ten robotics journals. The MCC is unknown probably also to the reviewers and the associate editors who handled the review of these manuscripts and did not invite the authors to include results measured by this statistical rate.

All the authors of all the manuscripts published in five robotics journals (*Frontiers in Robotics and AI*, *IEEE Robotics and Automation Letters*, *IEEE Transactions on Robotics*, *Robotics and Intelligent Systems*, and *Science Robotics*) decided not to include any result measured by the MCC.

Regarding *Frontiers in Artificial Intelligence*, we notice that the Matthews correlation coefficient was employed by the authors of three original research studies (Bhatt et al., 2021; Li et al., 2021; Wu et al., 2021), two methods articles (Fletcher et al., 2021; Weerawardhana et al., 2022), and one review (Tripathi et al., 2021). The study of Li et al. (2021) presents a deep learning application on chemoinformatics data for the prediction of carcinogenicity. Chemical data analysis is also the topic of the article by Wu et al. (2021), which employs natural language processing techniques for drug labeling and indexing. Fletcher et al. (2021), instead, present a study on fairness in artificial intelligence applied to public health, reporting a case study on machine learning applied to data of pulmonary disease. Weerawardhana et al. (2022) employed the MCC to measure the results in a human-aware intervention and behavior classification study, In their review article, Tripathi et al. (2021) reported some AI best practices in manufacturing, indicating the MCC as one of the confusion matrix rates employed in this field.

Among the five articles published in the *Robotics and Autonomous Systems* journal, three are about robots’ visual activities (Bosse and Zlot, 2009; Özbilge, 2016; Özbilge, 2019), one is about swarm robotics (Lau et al., 2011), and one is about human–robot verbal interaction (Grassi et al., 2022).

The only article of *Frontiers in Neurorobotics* including results measured by the MCC is a study on visual perception of robots (Layher et al., 2017), while the only MCC study in *International Journal of Robotics Research* describes a dataset on urban point cloud obtained acquired by mobile laser scanning (Roynard et al., 2018). The article of the *Journal of Field Robotics* including MCC results is about the robotics visual obstacle detection (Santana et al., 2011). The presence of the MCC in these studies does not seem to follow a precise trend, but rather be occasionally employed by authors who are aware of MCC’s assets, for reasons we do not know.

Regarding dates, it is interesting to notice that, except one article published in 2009 and one in 2011, all the other studies were published after 2016, showing an increased interest towards the Matthews correlation coefficient. Eight articles out of fourteen have been published in 2021 and 2022, suggesting a greater use of the MCC in future studies.

As we explained earlier, the amount of articles including MCC results is very low compared to the number of published studies involving accuracy and F_1_ score (Table 1). And we think this is a serious drawback: as we explained in our study (Chicco and Jurman, 2020), the Matthews correlation coefficient is more informative and reliable than accuracy and F_1_ score, because it takes into account the ratio of positive data instances, negative data instances, positive predictions, and negative predictions.

Accuracy and F_1_ score both range between 0 and 1, with 0 meaning worst result possible and 1 meaning perfect prediction. An accuracy value of 0.9 and a F_1_ score of 0.95, for example, suggest a very good binary classification. If the original dataset consisted of 91 positive elements and 9 negative elements, these results could be generated by a cracked classifier that labels everything as positive. If a classifier assigned the “positive” label to all the 100 data elements, the evaluation procedure would get accuracy = 0.9 and F_1_  score = 0.95, which are clearly misleading results and could let the practitioner think that the binary classification was excellent. The MCC, instead, would have been –0.03, that in the [ − 1, +1] interval indicates a poor prediction similar to random guessing: the MCC would inform the practitioner that her/his binary classification was quite bad, while accuracy and F_1_ score tried to make her/him believe it was great.

We therefore invite the robotics and artificial intelligence communities to include results measured through the MCC for any binary classification analysis.
